# Dr. A. Thos. Houghton Waters

**Published:** 1912-06-29

**Authors:** 


### Obituary.

Dr. A. Thos. Houghton Waters has died at his residence in Liverpool at the age of eighty-six. He qualified in 1853 as M.R.C.S. (Eng.), and was the oldest member of the profession in Liverpool. Dr. Waters was formerly Professor of the Principles and Practice of Medicine at the University of Liverpool, towards the establishment of which he did some very hard pioneer work in the seventies. In 1861 he took the M.D. of St. Andrews, and in 1867 was elected a Fellow of the Royal College of Physicians of London. His academic successes were numerous, and included the Fothergill gold medal for an essay on the anatomy of the lung. Dr. Waters contributed very largely to medical literature, paying special attention to the diseases of the thoracic organs and to tuberculosis. Of his hospital appointments, the chief were honorary physician to the Northern Hospital and the Liverpool Royal Infirmary, to this latter he was consulting physician at the time of his death. When the British Medical Association met in Liverpool in 1883 Dr. Waters became the President.

